# Identification of Auxiliary Biomarkers and Description of the Immune Microenvironmental Characteristics in Duchenne Muscular Dystrophy by Bioinformatical Analysis and Experiment

**DOI:** 10.3389/fnins.2022.891670

**Published:** 2022-06-03

**Authors:** Xu Han, Jingzhe Han, Ning Wang, Guang Ji, Ruoyi Guo, Jing Li, Hongran Wu, Shaojuan Ma, Pingping Fang, Xueqin Song

**Affiliations:** ^1^Department of Neurology, The Second Hospital of Hebei Medical University, Shijiazhuang, China; ^2^Neurological Laboratory of Hebei Province, Shijiazhuang, China; ^3^Department of Neurology, Handan Central Hospital, Handan, China

**Keywords:** Duchenne muscular dystrophy, diagnostic biomarkers, immune microenvironment, bioinformatical analysis, RT-qPCR

## Abstract

**Background:**

Duchenne muscular dystrophy (DMD) is a genetic muscle disorder characterized by progressive muscle wasting associated with persistent inflammation. In this study, we aimed to identify auxiliary biomarkers and further characterize the immune microenvironment in DMD.

**Methods:**

Differentially expressed genes (DEGs) were identified between DMD and normal muscle tissues based on Gene Expression Omnibus (GEO) datasets. Bioinformatical analysis was used to screen and identify potential diagnostic signatures of DMD which were further validated by real-time quantitative reverse transcription PCR (RT-qPCR). We also performed single-sample gene-set enrichment analysis (ssGSEA) to characterize the proportion of tissue-infiltrating immune cells to determine the inflammatory state of DMD.

**Results:**

In total, 182 downregulated genes and 263 upregulated genes were identified in DMD. *C3, SPP1, TMSB10, TYROBP* were regarded as adjunct biomarkers and successfully validated by RT-qPCR. The infiltration of macrophages, CD4+, and CD8+ T cells was significantly higher (*p* < 0.05) in DMD compared with normal muscle tissues, while the infiltration of activated B cells, CD56dim natural killer cells, and type 17 T helper (Th17) cells was lower. In addition, the four biomarkers (*C3, SPP1, TMSB10, TYROBP*) were strongly associated with immune cells and immune-related pathways in DMD muscle tissues.

**Conclusion:**

Analyses demonstrated *C3, SPP1, TMSB10*, and *TYROBP* may serve as biomarkers and enhance our understanding of immune responses in DMD. The infiltration of immune cells into the muscle microenvironment might exert a critical impact on the development and occurrence of DMD.

## Introduction

Duchenne Muscular Dystrophy (DMD), also referred to as dystrophinopathy, is a devastating X-linked disease caused by mutations in the dystrophin gene which affects 1 in 5,000 to 1 in 6,000 live male births (Duan et al., 2021). Dystrophin acts as a bridge between the cytoskeleton and the extracellular matrix in skeletal and cardiac muscle fibers. The absence of dystrophin destabilizes cell membranes and causes sarcolemmal fragility, cell necrosis and inflammatory responses. The regenerative capacity of the muscle fibers is impaired in a chronic inflammatory environment and necrotic muscle cells are eventually replaced by fat and fibrotic tissue. Most patients with DMD are wheelchair-bound in their early teens, require ventilatory support in their late teens, and eventually die in their early twenties from cardiac/respiratory failure (Rosenberg et al., 2015). Consequently, there is an urgent need to improve therapeutic strategies for patients with DMD. However, current treatments mainly rely on steroid-based therapy, non-sense readthrough, and exon skipping, which have limited efficacy, short duration effects, and various adverse effects (Shieh, 2018). Some promising treatments, such as genome editing and stem cell treatment, are limited by the immune response of the human body (Maffioletti et al., 2014; Duan et al., 2021; Hakim et al., 2021). Therapies to attenuate the inflammatory response in the muscle microenvironment could be an important and effective approach to prevent degeneration and adjunctive therapy to other emerging treatments aiming to repair the muscle (Mendell et al., 2010; Cordova et al., 2018). Therefore, it is vital to further characterize the immune microenvironment and identify novel therapies for DMD.

Previous studies have shown the infiltration of immune cells plays a crucial role in the progression of DMD. Initial fiber necrosis caused by sarcolemmal instability contributes to a severe inflammatory response, including the infiltration of immune cells and the increased secretion of proinflammatory cytokines, thus leading to a 400–500% increase in damage to the membranes of muscle cells (Porter et al., 2002; Tidball et al., 2018). For example, infiltrating M1 macrophages can promote the lysis of muscle cells *via* the expression and secretion of various pro-inflammatory cytokines. M2 macrophages are thought to be essential partners in muscle regeneration but may also promote myocardial and skeletal muscle fibrosis (Deconinck and Dan, 2007; Perandini et al., 2018). CD8+ T cells can directly destroy muscle fibers *via* perforin-mediated cytotoxicity while CD4+ T cells can enhance the cytocidal effects of CD8+ T cells and macrophage activity by secreting cytokines (Spencer et al., 2001). However, how immune cells affect the pathological progression of DMD needs further study. Evaluating the infiltration of immune cells and identifying the differences between the components of infiltrating immune cells are critical to fully elucidate the biological mechanisms underlying DMD and identify new immunotherapeutic targets.

Advances in genome sequencing technologies and the microarray analysis of disease-related datasets have made it possible for us to quantify immune cell infiltration in a diverse set of different diseases. Over recent years, several studies have provided an overview of the immune response as a key molecular pathway in DMD and identified key genes by using Gene Expression Omnibus (GEO) datasets (Wang et al., 2021; Xu et al., 2021). However, the specific relationships between immune cells and the key genes which influence inflammatory responses have yet to be studied systematically; the differences in the expression of key genes between patients with DMD and normal individuals remain unclear.

In the present study, bioinformatic analyses were applied to identify auxiliary biomarkers of DMD and the result was validated by real-time quantitative reverse transcription PCR (RT-qPCR). To investigate the infiltration of immune cells in DMD, we performed the single-sample gene set enrichment analysis (ssGSEA) and found the effects of immune-related genes on the main immune-related pathways by using gene set variation analysis (GSVA). Our findings emphasize the association between key genes and the immune microenvironment at the molecular level and provide new immunotherapeutic targets for patients with DMD.

## Materials and Methods

### Data Collection and Data Processing

Microarray datasets for gene expression analyses were downloaded from the GEO database (GEO^1^), including GSE6011 (23 DMD muscle tissues and 14 normal muscle tissues) (Pescatori et al., 2007), GSE38417 (16 DMD muscle tissues and 6 normal muscle tissues) (Khairallah et al., 2012), and GSE109178 (17 DMD muscle tissues and 6 normal muscle tissues) (Dadgar et al., 2014; Dang et al., 2020). The GSE6011 dataset is based on the Affymetrix GPL96 platform, while the GSE38417 and GSE109178 datasets are based on the Affymetrix GPL570 platform (Table 1). The probe ID was then mapped to the appropriate gene symbol and the gene expression value was averaged if multiple probes were associated with the same gene symbol.

**TABLE 1:** Information for GSE6011, GSE38417, and GSE109178.

GEO accession	Platform	Samples		Source tissue	Age (Mean ± SEM)		Sex (male/female)		Country
					
		DMD	Control		DMD	Control	DMD	Control	
GSE6011	GPL96	23	14	Skeletal muscle	1.24±0.26	2.14±0.67	23/0	11/3	Netherlands
GSE38417	GPL570	16	6	Skeletal muscle	3.76±0.52	N/A	16/0	N/A	United States
GSE109178	GPL570	17	6	Skeletal muscle	3.75±0.53	N/A	17/0	N/A	United States

### Data Normalization and Identification of Differentially Expressed Genes

Heterogeneity and latent variables are often considered to be the main sources of bias and variability in high-throughput experiments. We normalized the included GEO datasets to render the sample genes in each dataset comparable by the “LIMMA” R package. In addition, the small sample size of the dataset may affect the power of the statistical analysis and introduce certain inaccuracies to the results. Therefore, we integrated all samples from the three datasets to expand the sample size (26 normal samples *vs.* 56 DMD samples) and eliminated the batch effect by applying the combat algorithm in the “sav” R package. Data analysis on the standardized, normalized and merged dataset was performed by the “LIMMA” R package. A *p*-value < 0.05 and a log2 (fold change) criterion > 2 were used to identify differentially expressed genes (DEGs). To better visualize these DEGs, volcano plots were generated using *R* software and heatmap of the first 30 DEGs was produced by the heatmap package.

### Construction of Protein-Protein Interaction Network and Biological Pathway Analysis

The Search Tool for the Retrieval of Interacting Genes/Proteins (STRING^2^) is a tool which quantitatively integrates interaction data, conserved gene co-expression, genomic context and high-throughput experiments. In this study, a protein-protein interaction (PPI) network of DEGs was created using the STRING tool. To insight into the functions of the DEGs and identify the significant pathways associated with the DEGs, the Gene Ontology (GO) analyses and Kyoto Encyclopedia of Genes and Genomes (KEGG) analysis were performed with the “clusterProfiler” package in R. An enrichment *p*-value < 0.05 was statistically significant.

### Analysis of Microarray Datasets for Immune Cell Infiltration

The DEGs were intersected with immune-related genes^3^ and these overlapping genes were defined as immune-related DEGs and visualized by Venn graphs. The ssGSEA algorithm was performed to quantify the relative extent of infiltration for each immune cell in DMD and normal muscle tissues. The data from the previous study relating to various human immune cell subtypes, including macrophages, activated dendritic cells, natural killer T cells, and activated CD4+ T cells (Charoentong et al., 2017), was applied to derive the marker gene set for each type of infiltrating immune cell. The enrichment scores calculated by ssGSEA analysis were used to represent the relative abundance of each DMD infiltrating cell in each sample.

### LASSO Regression Analysis and ROC Curve Assessment

To identify the signatures in the high-dimensional data, a LASSO model was established based on the gene expression profiles of immune-related DEGs by the “glmnet” package in R. The best variable in the LASSO model was determined according to the minimum λ value. We conducted regression analysis for the genes acquired from the LASSO model and calculated the regression coefficient of candidate genes according to the following formula:


$$\mathrm{Index}=\sum_{i=1}^{n}{\mathrm{Expri}}^{*}\,\mathrm{Coefi}$$

where “Expri” represents the expression value of a gene and “Coefi” represents the regression coefficient of a gene. In addition, the combined samples were randomly split to training and validation datasets in a 1:1 ratio and ROC curve analysis was performed to evaluate stability and sensitivity of the model by “pROC” R package.

### Gene Set Variation Analysis

Gene set variation analysis (GSVA) is a non-parametric and unsupervised gene set enrichment tool which can evaluate the association between gene signatures and biological pathways according to expression profile data (Hanzelmann et al., 2013). By downloading the “h.all. v7.2. symbols” gene set from the Molecular Signatures Database (MSigDB), we analyzed the main immune-related pathways which might be associated with our four candidate genes. Results arising from the GSVA were visualized as a heat map.

### Patient Samples

All included patients had a diagnostic confirmation of DMD at the Second Hospital of Hebei Medical University between July 2020 and June 2021 by *DMD* mutation and the absence of dystrophin expression on muscle biopsy. Concurrently, muscle biopsy specimens with normal histology and immunohistochemistry were collected as the control group. Clinical information is summarized in Table 2. This study was ethically approved by the Research Ethics Committee of the Second Hospital of Hebei Medical University (Shijiazhuang, China) and all participants provided informed consent in a written form prior to research. The fresh muscular samples were frozen in liquid nitrogen and stored in the RNA stabilization reagent, RNAlater (Beyotime Institute of Biotechnology, Nantong, China) at −80°C to prevent RNA from degradation. The sample size was chosen according to Emilie Vénéreau et al’s study (Careccia et al., 2021) and the experiment was terminated after analyzing five muscle specimens of DMD patients because a clear statistical difference was observed compared with healthy controls (*n* = 5).

**TABLE 2:** Clinical background information of the human participants.

Sample	Sex	Age	Source tissue	Dystrophin protein expression	Genetic data (when available)
DMD 1	Male	8	Skeletal muscle	Absent	Exon 49–52 deletions
DMD 2	Male	10	Skeletal muscle	Absent	Exon 51–59 duplication
DMD 3	Male	11	Skeletal muscle	Absent	Point Mutation
DMD 4	Male	8	Skeletal muscle	Absent	Exon 49–52 deletions
DMD 5	Male	9	Skeletal muscle	Absent	Point Mutation
Control 1	Male	17	Skeletal muscle	Normal	–
Control 2	Male	14	Skeletal muscle	Normal	–
Control 3	Male	12	Skeletal muscle	Normal	–
Control 4	Male	6	Skeletal muscle	Normal	–
Control 5	Male	15	Skeletal muscle	Normal	–

### Real-Time Quantitative Reverse Transcription PCR

We extracted total RNA with Trizol reagent (Servicebio, Wuhan, China) and measured the concentration by Nanodrop. Then the total RNA was reverse transcribed with the cDNA Reverse Transcription Kit (Servicebio, Wuhan, China). Gene expression level was determined by SYBRGreen (Vazyme Biotech, Nanjing, China). All primers (Table 3) were synthesized by Sangon Biotech (Shanghai, China). Relative expression values were calculated by using the 2-ΔΔCT method. Referring to the reference genes used in the previous study (De Pasquale et al., 2012; Hildyard et al., 2018; Alonso-Jimenez et al., 2021), we chose *GAPDH, SDHA, HPRT1 and RPL13a* as possible reference genes and determined the amount of their expressions in DMD and control groups by RT-qPCR. The web-based tool RefFinder^4^ (Lu et al., 2018) was used to comprehensively evaluated the stability of candidate reference genes and *GAPDH* was shown as the most stable gene (Supplementary Figure 1), which was then chosen as an internal control.

**TABLE 3:** The primer sequences used in the study.

Gene	Accession number	Forward primer	Reverse primer
GAPDH	NM_001256799.3	GGAAGCTTGTCATCAATGGAAATC	TGATGACCCTTTTGGCTCCC
C3	NM_000064.4	CACCGACTTCATCCCTTCCTT	CCGTGGTCACCCTCTATCTTCA
Spp1	NM_000582.3	CAGCCGTGGGAAGGACAGTTATG	TCACATCGGAATGCTCATTGCTCTC
TMSB10	NM_021103.4	ACAAACCAGACATGGGGGAAA	CTCAATGGTCTCTTTGGTCGG
TYROBP	NM_001173514.2	ACTGAGACCGAGTCGCCTTATC	GATGGCACTCTGTGGGTCTGTAT

### Statistical Analysis

Correlation analyses between the expression levels of key genes and infiltrating immune cells were performed by Spearman’s correlation and distance correlation analyses. Differences in the continuous variables between the two groups were compared by Student’s *t*-test or Mann–Whitney *U*-test. Statistical analyses were performed by one-way analysis of variance (ANOVA) or Kruskal–Wallis test for the comparison of continuous variables between three or more groups. The diagnostic values of gene expressions were analyzed by ROC curve analyses and estimated by the area under the curve (AUC) analysis. Statistical significance was declared at *p* < 0.05 and all *P* values were two-sided. All data processing was carried out with R version 3.6.1 software and SPSS version 21.0 software.

## Results

### Identification of Differentially Expressed Genes

**FIGURE 1:**
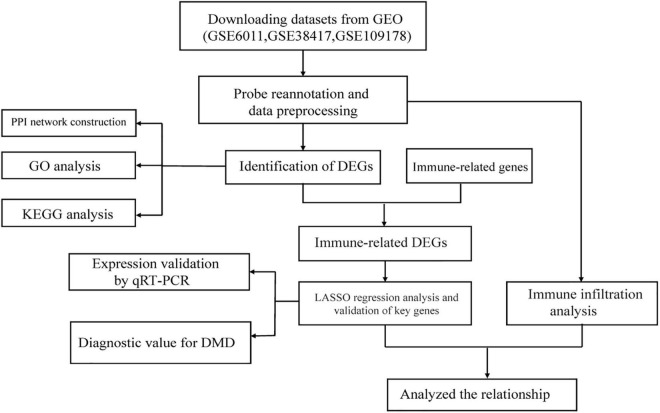
Schematic illustration of the workflow involved in this study.

**FIGURE 2:**
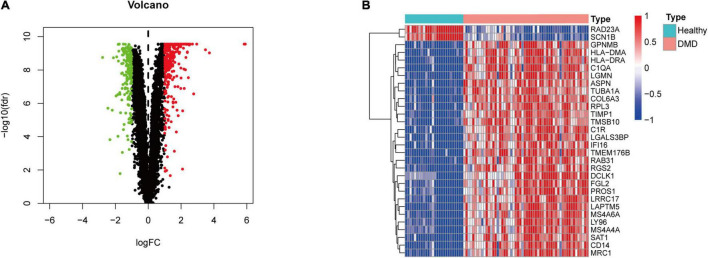
Differential gene expression analysis. **(A)** Volcano plot representation of differentially expressed genes (DEGs) identified between DMD samples and normal samples. Red, green, and black plots indicate upregulated, downregulated, and non-significant genes, respectively, where the ordinate is -log10 (FDR) and the abscissa is logFC. **(B)** Heat map based on the first 30 DEGs. Red rectangles indicate high expression while green rectangles indicate low expression.

Figure 1 illustrates the workflow of this study. First, we normalized the data in the three datasets (Supplementary Figure 2) and then integrated the datasets. In total, 82 samples (26 normal samples and 56 DMD samples) were extracted from the three datasets. Next, we applied combat algorithm to eliminate batch effect and principal component analysis (PCA) to visualize data grouping (Supplementary Figure 3). As shown in the volcano plot (Figure 2A), 445 DEGs were identified in DMD muscle tissues compared with normal muscle tissues, including 182 downregulated genes and 263 upregulated genes. The heat map indicated that expression levels of the first 30 DEGs showed significant differences between the DMD group and the non-muscular dystrophy group (Figure 2B).

### Protein-Protein Interaction Network Analysis and Functional Analyses

**FIGURE 3:**
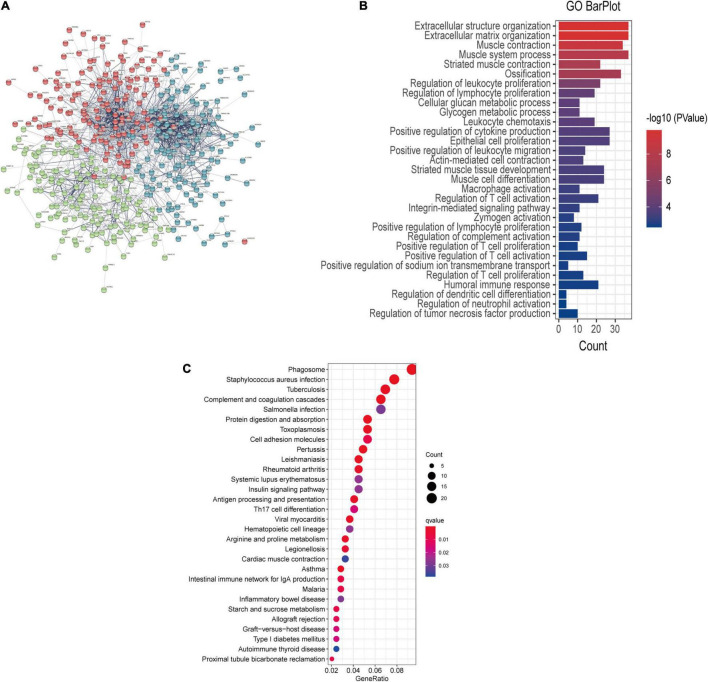
**(A)** PPI network showing the interactions of the DEGs. The thickness of the line in the figure designates the strength of the correlation. **(B)** GO enrichment analysis and **(C)** KEGG analysis of the DEGs.

The interaction network between proteins was generated from DEGs based on STRING and visualized by Cytoscape software. There was a significant association between the proteins encoded by the 445 genes (Figure 3A). GO functional enrichment analysis reveals the biological pathways associated with these genes and demonstrated that there was a strong association between DEGs and the extracellular matrix and immune signaling pathways, including extracellular matrix organization, humoral immune response, the regulation of lymphocyte proliferation, and epithelial cell proliferation (Figure 3B and Supplementary Table 1). KEGG analysis further revealed that the complement and coagulation cascades were the most enriched pathways (Figure 3C).

### Identification of Candidate Immune-Related Genes and the Landscape of Immune Infiltration in Duchenne Muscular Dystrophy

**FIGURE 4:**
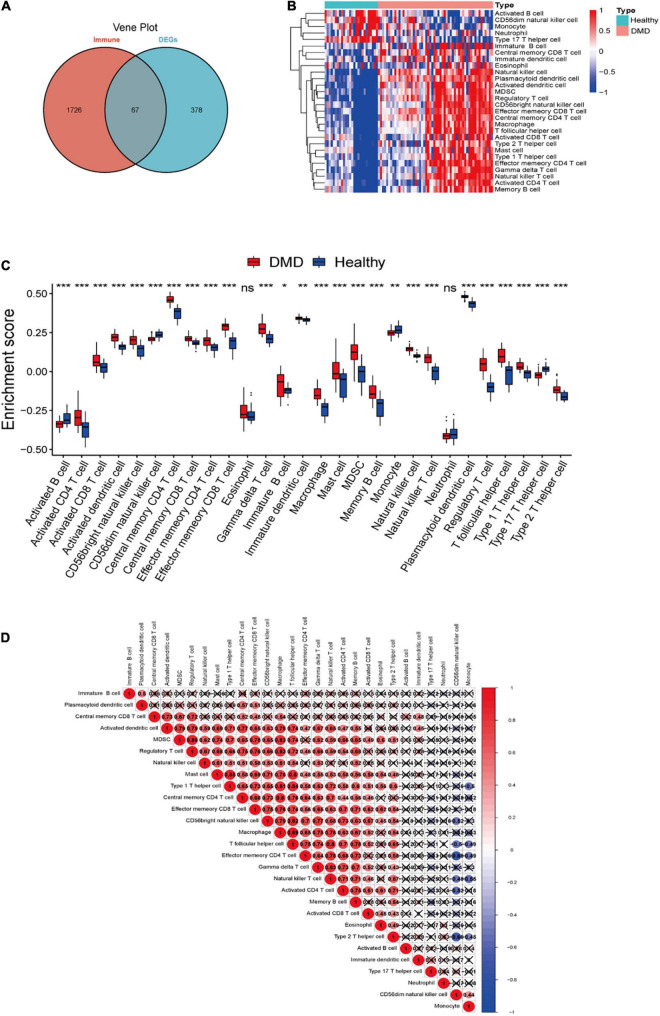
**(A)** Venn diagram showing the identification of DEGs which were correlated with immune cells in DMD. **(B)** The overall landscape of immune cell infiltration in DMD (*n* = 56 tissues). **(C)** Box plot showing the scores for 28 immune cells. **(D)** Correlations between 28 immune cells infiltrating in DMD muscle tissues (red for positive correlation, blue for negative correlation, white for weak or no correlation). ns, not significant; *P < 0.05; **P < 0.01; ***P < 0.001.

Totally 67 immune-related DEGs were identified by overlapping DEGs with immune-related genes (Figure 4A and Supplementary Table 2). Our data showed that the pathological process of DMD might be related to immunity. The landscape of 28 immune cell types in DMD was evaluated by ssGSEA and the result suggested that the proportion of different immune cells showed notable differences between DMD and normal groups. Specially, macrophages, CD56bright natural killer cells, CD4+ cells and CD8+ T cells were significantly upregulated (*P* < 0.05) in DMD tissues, while type 17 T helper cells, activated B cells, and CD56dim natural killer cells showed lower levels of expression (Figures 4B,C). Furthermore, we analyzed the association of 28 immune cell subpopulations in DMD tissues and identified a positive relationship between these upregulated immune cell types (Figure 4D).

### LASSO Model Construction and the Validation of Key Genes

**FIGURE 5:**
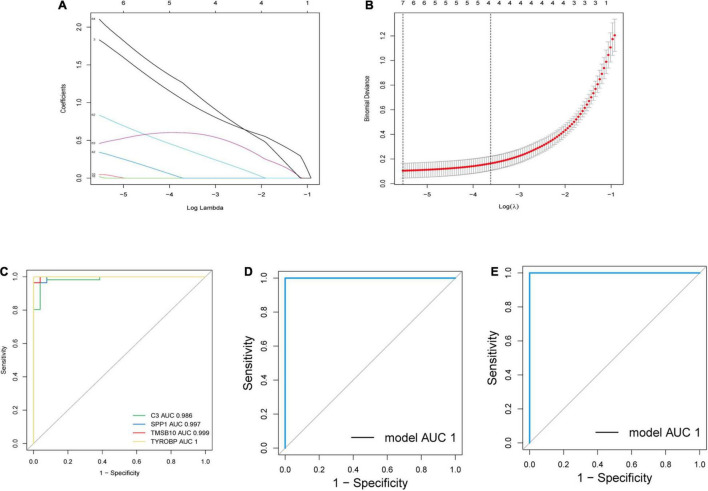
Establishment and verification of a LASSO model. **(A)** LASSO coefficient profiles of a model featuring the selected four genes. **(B)** Plots of 10-fold cross-validation error rates. **(C)** ROC curves for the four candidate genes. **(D,E)** ROC curves for the four-gene-model in the training dataset and validation dataset. *AUC*, area under the ROC curve.

We extracted the expression profiles of the identified immune-related genes and established a LASSO model (Figures 5A,B). In total, 67 immune-related genes were analyzed further by LASSO regression based on our minimum criteria; four genes (*C3*, *SPP1*, *TMSB10*, and *TYROBP*) were finally selected to construct gene signatures. The four candidate genes were identified according to the model index based on the following formula: Index = (1.0064) × *C3* + (0.3751) × *SPP1* + (0.5989) × *TMSB10* + (1.2125) × *TYROBP*. The accuracy of the model was assessed by ROC curves and predicted by AUC values. As shown in Figure 5C, the AUCs for *C3, SPP1, TMSB10*, and *TYROBP* were 0.986, 0.997, 0.999, and 1, respectively, which indicates a high accuracy of our model. Then, by randomly dividing all the samples into a training dataset and a validation dataset, we found that the AUC was 1 in both datasets (Figures 5D,E). Therefore, these genes have the potential to serve as auxiliary diagnostic biomarkers for further analyses.

**FIGURE 6:**
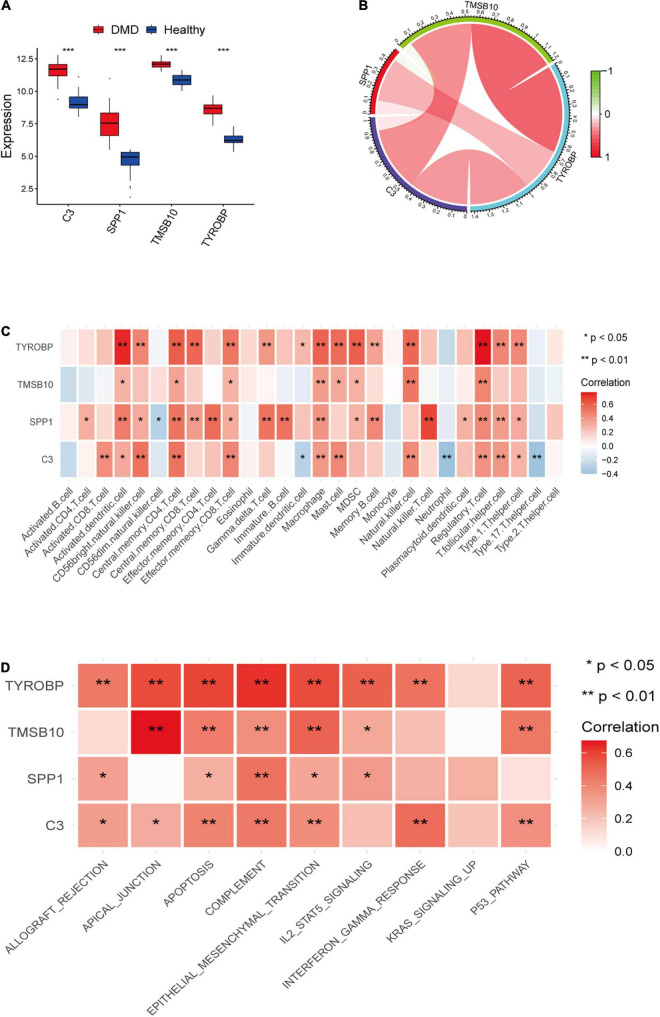
**(A)** Box plot of the expression differences of candidate genes between the DMD group and healthy control group. **(B)** A map of expression correlation between candidate genes in the model; red indicates a positive correlation while blue indicates a negative correlation; the darker the color, the stronger the correlation. **(C)** Heatmap showing correlation analysis between candidate genes and 28 immune cells. **(D)** Heatmap showing the relationship between candidate genes and potential immune pathways. Red represents a positive correlation. *P < 0.05; **P < 0.01; ***P < 0.001.

We further analyzed the expression levels of four candidate genes verified by LASSO regression in DMD and normal muscle tissues and presented the results as boxplots (Figure 6A). Compared with normal muscle tissues, the expression levels of *C3, SPP1, TMSB10*, and *TYROBP* were significantly higher (*P* < 0.05) in DMD. We checked correlations between the four candidate genes and found the expression levels of *C3*, *TMSB10*, and *TYROBP* were positively correlated (Figure 6B). Then, the relationship between infiltrating immune cells and the expression levels of candidate genes was explored. As indicated in the heat map (Figure 6C), a significant association was found in the muscle tissues of DMD patients. Furthermore, the main immune-related pathways associated with the four candidate genes were investigated by GSVA (Figure 6D). The result suggested that the target genes might take part in many important immune signaling pathways, such as the complement, IL2-STAT5 signaling pathways and the response to interferon gamma.

**FIGURE 7:**
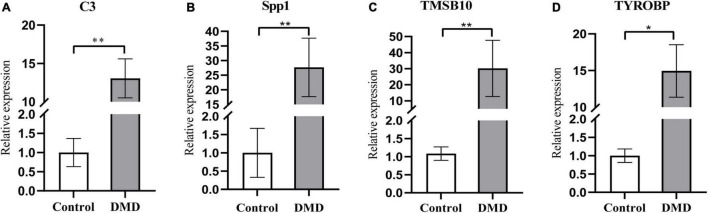
The relative mRNA expression levels of *C3* **(A)**, *Spp1* **(B)**, *TMSB10* **(C)**, and *TYROBP* **(D)** in muscle tissues from DMD and control groups, as detected by RT-qPCR with *GAPDH* as the reference gene. *P < 0.05; **P < 0.01.

The expression levels of the four candidate genes were validated by RT-qPCR in five patients with DMD and five healthy controls. The expression levels of *C3*, *SPP1*, *TMSB10* and *TYROBP* were significantly upregulated in the muscle tissues of DMD compared with control group (*P* < 0.05), as detected by RT-qPCR with *GAPDH* as the reference gene (Figure 7). Furthermore, *SDHA, RPL13a* were used as the internal controls for further validation and the results were consistent with the data by using GAPDH as a reference gene (Supplementary Figure 4), which demonstrate the reproducibility and reliability of our analyses.

## Discussion

Duchenne muscular dystrophy is a genetic disease characterized by progressive muscle wasting and persistent inflammation. Although mechanical damage and cell membrane defects are known to be basic pathological changes in DMD, the infiltration of immune cells also has a significant impact on the occurrence and progression of DMD (Tidball et al., 2018). In particular, the immune environment of the muscle tissue in DMD patients is known to restrict the therapeutic effects of some promising treatments, such as gene editing and stem cell transplantation (Duan et al., 2021; Tulangekar and Sztal, 2021). Therefore, exploring potential diagnostic biomarkers and characterizing the infiltration pattern of immune cells in DMD is of great significance to enhance the diagnosis and prognosis of DMD (Mendell et al., 2010).

In the present study, we sought to identify auxiliary diagnostic markers related to DMD immunity and analyze their relationship with immune cell infiltration in DMD. First, we identified 445 DEGs between DMD and normal muscle tissues. Functional enrichment analysis demonstrated that the DEGs were closely related to immune response and inflammatory signaling pathways, thus demonstrating DMD has a strong immune activation process which may lead to the inflammatory process in muscle tissue, thereby inducing muscular necrosis and atrophy. We also identified four immune-related DEGs (*C3*, *SPP1*, *TMSB10*, and *TYROBP*) as candidate genes by LASSO regression; ROC curves showed the performance of the LASSO model was superior. Finally, we found that the infiltration of immune cells into the muscle microenvironment might be critical in the occurrence and development of DMD.

Complement C3 is a central protein of the complement system which plays a core role in the activation of the complement system (Dalakas et al., 2020). A previous study reported the deposition of C3 and C9 in both necrotic and non-necrotic fibers of dystrophic muscles following pathological examination of muscle biopsies (Spuler and Engel, 1998). Moreover, Hodgetts and Grounds (2001) reported the depletion of C3 increased initial donor male myoblast survival in murine models. Another study confirmed that dysferlin-deficient mice with C3 knockout exhibited a mitigating effect on muscle pathology. Unexpectedly, the ablation of C3 did not affect muscle pathology in the murine mdx model of DMD (Han et al., 2010), which may reflect the lower degree of muscle pathology in mdx mice than that in DMD patients or may indicate species-specific differences in complement activity. Therefore, further studies are needed to validate the feasibility of C3 as a biomarker of DMD and its role as a potential therapeutic target in DMD. Secreted phosphoprotein 1 (Spp1; osteopontin or micropontonin), which enhances extracellular matrix synthesis and turnover, is significantly elevated in DMD and mdx limbs (Haslett et al., 2002; Porter et al., 2002). A study showed that *Spp1* contributed to the aggravation of inflammatory-dependent pathology in the mdx model by skewing the polarization of macrophages and promoting fibrosis. The genetic deletion of *Spp1* in mdx mice presented with milder pathology, a greater contractile force in the muscle, and less fibrosis compared with control mdx littermates (Vetrone et al., 2009). Studies have also demonstrated that single nucleotide polymorphisms (SNPs) of *SPP1* loci may be associated with disease severity in DMD patients (Bello et al., 2012; Hoffman et al., 2013). Furthermore, as a genetic modifier, *Spp1* has been shown to have a direct role in the regulation of myofiber damage and may serve as a novel therapeutic option for dystrophic muscle (Quattrocelli et al., 2017). Collectively, these studies implicated the profound effect of Spp1 protein and its gene upregulation in DMD. Thymosin beta 10 (TMSB10), a protein localized to the cytoskeleton, is thought to enable actin monomer binding activity and participates in the regulation of cell migration. Although many studies have supported that TMSB10 promotes macrophage M2 conversion and serves as a biomarker in cases with malignant tumors (Bouchal et al., 2015; Zeng et al., 2020; Yan et al., 2021), few studies have investigated its specific role in DMD cases. The *TYROBP* gene, also known as *DAP12*, serves as a downstream adaptor for many immune receptors such as trigger receptor expression myeloid 1 (TREM1) (Bouchon et al., 2000), trigger receptor expression myeloid 2 (TREM2) (Colonna, 2003), and complement receptor 3 (CR3) (Linnartz and Neumann, 2013). As a key component in the activation of signal transduction, TYROBP is mainly expressed in macrophages and NK cells (Lanier et al., 1998; Bostanci et al., 2011). The Toll-like receptors (TLRs), which are highly expressed in macrophages and skeletal muscle cells, TLR2/4 and TLR7/8/9 in particular, are known to play a prominent role in the sustained chronic inflammation exhibited by the muscle tissue in DMD (De Paepe, 2020). It has also been reported TLR4 can amplify the role of the NF-κB pathway *via* TREM1/TYROBP after binding to antigen (Campanholle et al., 2013; Pelham and Agrawal, 2014; Liu et al., 2019). Abnormal activation of the NF-κB signaling pathway contributes to the persistence of inflammation and skeletal muscle wasting during the course of DMD (Li et al., 2008). Therefore, we speculate TYROBP may be involved in the progression of DMD by mediating key inflammatory pathways. Recent studies have also identified *TYROBP* as a key gene in DMD co-expression networks based on other DMD datasets; these findings are consistent with those reported herein (Wang et al., 2021; Xu et al., 2021). Thus, *C3, SPP1, TMSB10*, and *TYROBP* are likely to affect the progression of the inflammatory response in DMD and serve as auxiliary markers, although further clinical research is needed to validate the diagnostic significance of these candidates.

Next, we investigated differences in the proportion of immune infiltration cell types between DMD and normal muscle tissue by ssGSEA. Analysis revealed the increase of infiltration in 23 out of 28 different types of immune cells in DMD, including macrophages, activated dendritic cells, activated CD4+ T cells, activated CD8+ T cells, regulatory T cells (Tregs), and CD56bright natural killer cells. The levels of infiltration for active B cells, CD56dim natural killer cells and type 17 T helper cells were decreased in DMD. These differences might be associated with DMD occurrence and progression, which were in good agreement with those from previous studies (Tripodi et al., 2021) and further confirmed the importance of the immune response in the pathogenesis of DMD. It is generally known that macrophages are an essential component of human innate immunity, and these cells can also exert potential influence on the pathogenesis of DMD. Studies have shown the activation of two functional subsets of macrophages contributes to the physiopathology of DMD: the classically activated M1 (pro-inflammatory M1) and alternatively, activated M2 (anti-inflammatory M2) macrophages. The predominant molecular mechanisms underlying the infiltration of M1 macrophages include enhanced oxidative stress, the production of pro-inflammatory cytokines (TNF-α, IL-6, and IFN-γ) and the expression of fibrotic genes. M2 macrophages can secrete anti-inflammatory cytokines (such as IL-10, IL-4) and promote regeneration (Deconinck and Dan, 2007). However, activated *M2* macrophages have also been implicated in the pathogenic pro-fibrogenic response (Perandini et al., 2018). The interaction of persistently activated macrophages with the cytokines they produce leads to progressive necrosis in the muscle fibers, the accumulation of extracellular matrix, and the eventual replacement of muscle fibers by regenerated adipose and fibrous tissue throughout the course of the disease (Tidball and Villalta, 2010). Serving as professional antigen presenting cells (APCs), dendritic cells (DCs) are generally thought to represent a bridge which connects the innate and the adaptive immunity. It has been demonstrated that DCs can modulate the differentiation of T cells toward Th1, Th2, Th17 or regulatory T (Treg) cells and thus play a role in the dystrophic muscle environment (Lorant et al., 2018). In addition, DCs may regulate the pathways mediated by TLRs and recognize several cytokines and pro-inflammatory mediators which are highly expressed in patients with DMD (Madaro and Bouche, 2014). However, few studies have addressed the potential contribution of DCs to the progression of DMD pathology *via* TLR7-mediated signals or the modulation of TGF-β expression (Lemos et al., 2015). Adaptive immunity, predominantly involving T cells, has also been shown to play an important role in the occurrence of DMD (Spencer et al., 2001); the infiltration of B cells is much rarer (Peccate et al., 2016). Our present data supports this viewpoint. The infiltration of CD8+ T cells into DMD muscle is known to cause direct damage to the muscle fibers *via* perforin-mediated cytotoxicity. Furthermore, CD4+ T cells can differentiate further into Th1 cells and secrete a series of cytokines, including IL-6, TGF*-*β, and IFN-γ to enhance the cytotoxic effects of CD8+ T cells and the activity of macrophages, thus leading to further enhancement of the inflammatory response (Spencer et al., 2001). We also found that another class of T cells, Tregs, was increased in DMD muscle tissue. Previous studies have shown Tregs can mitigate both inflammatory responses and fibrotic processes in DMD muscle and promote myofiber regeneration through the activation of satellite cells (Villalta et al., 2014). Therefore, factors that promote and inhibit inflammatory responses co-exist in DMD muscle tissue, which reflects the complexity of the muscle immune environment. Our results indicate that Type17 T helper cells (Th17) are reduced in DMD. As another differentiated subtype of CD4+ T cells, Th17 cells mainly produce the cytokine interleukin-17 (IL-17) (Miossec and Kolls, 2012). However, few studies have focused on the specific relationship between the infiltration of Th17 cells and DMD. The role of Th17 in DMD progression requires further investigation. We observed reduced levels of infiltration of CD56dim natural killer cells in DMD muscle tissue, along with increased CD56bright natural killer cells. CD56dim NK cell subsets predominantly exert cytotoxic functions, while CD56bright NK cell subsets exert their effects by producing pro-inflammatory factors (Cooper et al., 2001). These findings suggested NK cells might enhance the inflammatory response by secreting pro-inflammatory cytokines in DMD patients. However, further research needs to address the specific mechanisms controlling the infiltration of immune cells in DMD.

Over recent decades, numerous studies have revealed the sustained infiltration of inflammatory cells in patients with DMD plays an important role in disease pathogenesis. Immune cells and immune-related genes may prove to be promising therapeutic targets (Iftikhar et al., 2021). Importantly, the clinical outcomes and therapeutic response of DMD are closely related to the infiltration of innate and adaptive immune cells (Lu et al., 2003; Park et al., 2008; Doudna, 2020; Hakim et al., 2021). A therapeutic strategy based on the characteristics of the immune response represents an attractive approach to estimating the clinical course and outcomes of DMD. By performing ssGSEA to analyze microarray data from DMD, researchers could know the specific relationships between different immune cell subpopulations, which enhanced our understanding of the immune environment in the muscles of patients with DMD.

Studying the correlation between immune cells and candidate genes, we found *C3*, *Spp1*, *TMSB10*, and *TYROBP* were strongly correlated with infiltrating immune cells. In addition, GSVA analysis indicated a close relationship between these genes and immune pathways. The mRNA expression levels of *C3*, *Spp1*, *TMSB10*, and *TYROBP* in DMD patients were significantly higher than that in healthy control. These results indicate that the four newly identified markers may serve as new immunotherapeutic targets for DMD. However, the specific mechanisms underlying the interaction between immune-related genes and immune cells require further investigation.

Although the current study included a relatively large sample size by integrating GEO datasets to characterize the immune microenvironment and successfully discovered four genes with potential diagnostic value for DMD, some limitations need to be considered. First, as the present study explored the infiltration of immune cells by ssGSEA, it is essential to verify our results by flow cytometry. Second, the interactions between immune-related genes, immune cell infiltration and potential immune pathways require an in-depth investigation. Third, the expression levels of *C3*, *SPP1*, *TMSB10* and *TYROBP* in the muscular tissues of DMD were validated by RT-qPCR in the study. Nevertheless, due to the relatively small number of muscle tissues available for study, the patients were not stratified by pathological stage. In addition, more reference genes are suggested to improve the accuracy of RT-qPCR data (Vandesompele et al., 2002). Further studies are warranted to validate the prognostic function of these genes. Large research studies, incorporating clinical information and functional evaluation are needed to test and validate these important findings.

## Conclusion

In the present study, we analyzed the overall landscape of immune cell infiltration in DMD and identified *C3*, *SPP1*, *TMSB10*, and *TYROBP* as auxiliary diagnostic biomarkers for DMD. Next, we found the expressions of these biomarkers were strongly correlated with infiltrating immune cells in DMD. Finally, we validated that the mRNA expression level of these genes was significantly higher in DMD compared with healthy controls by RT-qPCR. Overall, the infiltration of immune cells into the muscle microenvironment might exert a critical impact on the occurrence and development of DMD. Our findings may provide new immunotherapeutic targets and serve as an adjunct to other treatments to reduce the immune-related side effects of these treatments and improve efficacy.

## Data Availability Statement

The datasets presented in this study can be found in online repositories. The names of the repository/repositories and accession number(s) can be found in the article/Supplementary Material.

## Ethics Statement

The studies involving human participants were reviewed and approved by Research Ethics Committee of the Second Hospital of Hebei Medical University. Written informed consent to participate in this study was provided by the participants’ legal guardian/next of kin.

## Author Contributions

XH, JH, and XS: study concept and design. XH, NW, and RG: data collection and processing. HW and SM: clinical sample collection. XH, JL, and PF: experiment conduction. XH: original draft writing. XS, JH, and GJ: manuscript review and editing. XS: funding acquisition. All authors read and approved the submitted version.

## Conflict of Interest

The authors declare that the research was conducted in the absence of any commercial or financial relationships that could be construed as a potential conflict of interest.

## Publisher’s Note

All claims expressed in this article are solely those of the authors and do not necessarily represent those of their affiliated organizations, or those of the publisher, the editors and the reviewers. Any product that may be evaluated in this article, or claim that may be made by its manufacturer, is not guaranteed or endorsed by the publisher.

## Footnotes

1
http://www.ncbi.nlm.nih.gov/geo/

2
https://string-db.org

3
https://www.immport.org/

4
http://blooge.cn/RefFinder/
