# Supplementary figures and images for: Identification of Auxiliary Biomarkers and Description of the Immune Microenvironmental Characteristics in Duchenne Muscular Dystrophy by Bioinformatical Analysis and Experiment
Source: Front Neurosci. 2022 Jun 3;16:891670. doi: 10.3389/fnins.2022.891670 (PMC9204148; doi:10.3389/fnins.2022.891670)

Supplement Figure 1

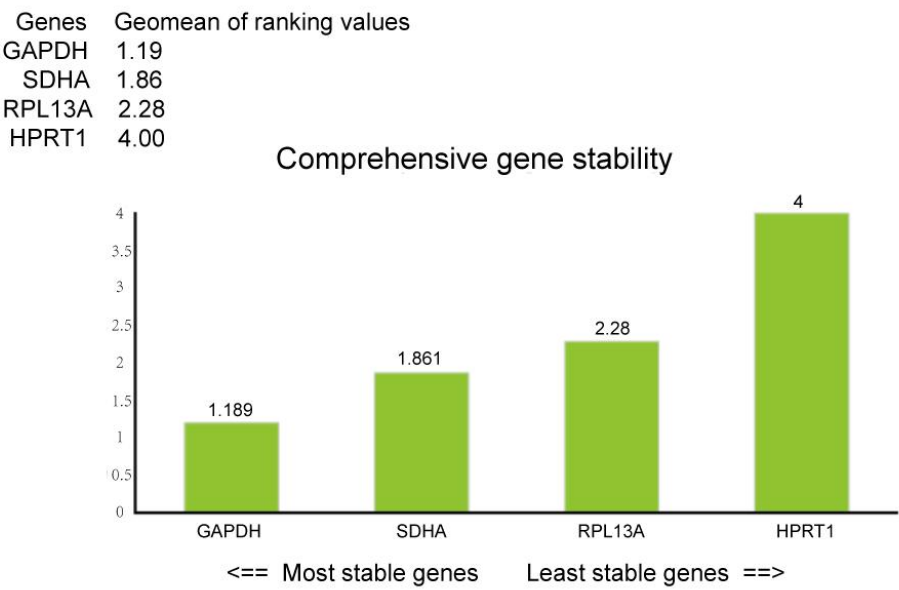

Figure S1. Gene expression stability ranked by RefFinder

Supplement: Supplementary file 1 [file Data_Sheet_1.pdf]
