# Supplementary material for: Identification of Auxiliary Biomarkers and Description of the Immune Microenvironmental Characteristics in Duchenne Muscular Dystrophy by Bioinformatical Analysis and Experiment
Source: Front Neurosci. 2022 Jun 3;16:891670. doi: 10.3389/fnins.2022.891670 (PMC9204148; doi:10.3389/fnins.2022.891670)
Supplement: Supplementary file 2 [file Data_Sheet_2.pdf]

Supplement Figure 2

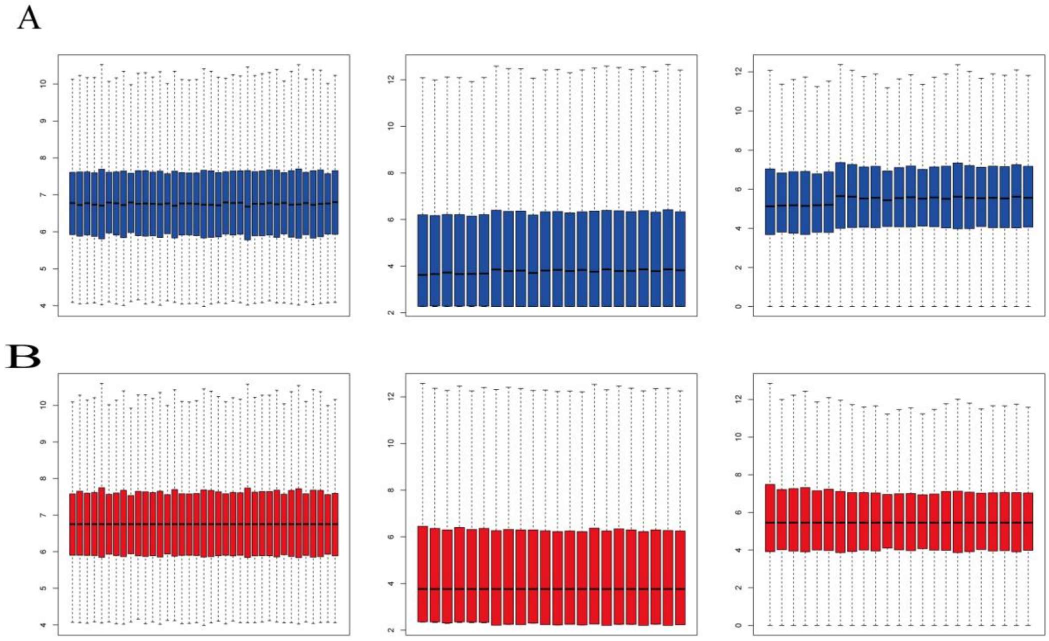

Figure S2. The boxplots for microarray data before (A) and after (B) normalization. Based on the standardization of the microarray data, the preprocessed data boxplots showed that the medians of each sample data were almost on the line, suggesting that the preprocessed data met the standard for further evaluation. The abscissa displays the samples, and the ordinate represents gene expression in each dataset (GSE6011, GSE38417, GSE109178).
