# Supplementary material for: Identification of Auxiliary Biomarkers and Description of the Immune Microenvironmental Characteristics in Duchenne Muscular Dystrophy by Bioinformatical Analysis and Experiment
Source: Front Neurosci. 2022 Jun 3;16:891670. doi: 10.3389/fnins.2022.891670 (PMC9204148; doi:10.3389/fnins.2022.891670)
Supplement: Supplementary file 3 [file Data_Sheet_3.pdf]

Supplement Figure 3

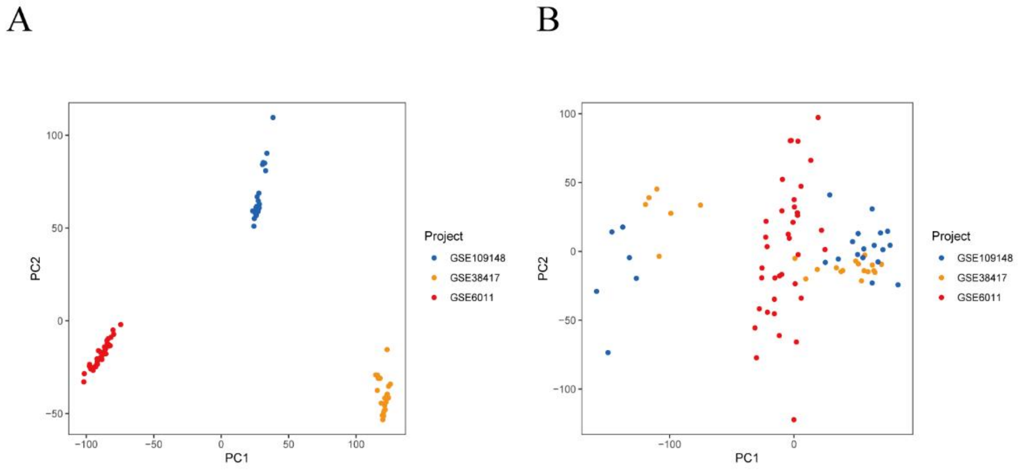

Figure S3. Presented the PCA is used to show data before (A) and after (B) using the Combat function to remove batch effects. (A) the distribution of data from the 3 datasets is relatively scattered, and the distance between the different datasets is relatively large. (B) The data between the datasets is relatively concentrated after batch effects are removed, which is beneficial for subsequent analysis.
