# Supplementary material for: Identification of Auxiliary Biomarkers and Description of the Immune Microenvironmental Characteristics in Duchenne Muscular Dystrophy by Bioinformatical Analysis and Experiment
Source: Front Neurosci. 2022 Jun 3;16:891670. doi: 10.3389/fnins.2022.891670 (PMC9204148; doi:10.3389/fnins.2022.891670)
Supplement: Supplementary file 4 [file Data_Sheet_4.PDF]

Supplement Figure 4

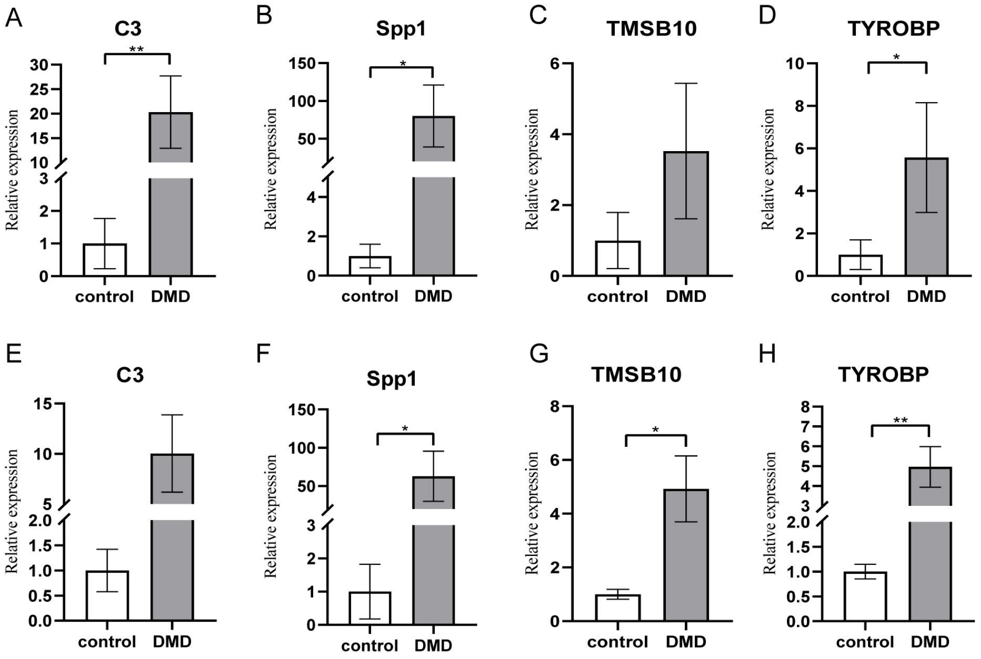

Figure S4. (A-D) *SDHA* was used as reference gene to normalize RT-qPCR results. The relative mRNA expression levels of *C3* (A), *Spp1* (B), *TYROBP* (D) were significantly unregulated in the muscular tissues of DMD compared with control group and *TMSB10* (C) showed a trend in increased expression (P=0.076). (E-H) *RPL13A* was used as reference gene. The relative mRNA expression levels of *Spp1* (F), *TMSB10*(G), *TYROBP* (H) were significantly unregulated in DMD compared with control group and *C3* (E) showed a trend in increased expression (P=0.095). *, P< 0.05; **, P< 0.01.
