# Supplementary material for: Identification of Auxiliary Biomarkers and Description of the Immune Microenvironmental Characteristics in Duchenne Muscular Dystrophy by Bioinformatical Analysis and Experiment
Source: Front Neurosci. 2022 Jun 3;16:891670. doi: 10.3389/fnins.2022.891670 (PMC9204148; doi:10.3389/fnins.2022.891670)
Supplement: Supplementary file 6 [file Table_2.docx]

| CD74 | CTSS | FCER1G | HLA-DMA | HLA-DMB | HLA-DPA1 | HLA-DPB1 |
| --- | --- | --- | --- | --- | --- | --- |
| HLA-DQA1 | HLA-DRA | HSPA2 | LGMN | PSMB8 | PROCR | CXCL14 |
| TMSB10 | PENK | S100A10 | S100A2 | S100A11 | S100A1 | COLEC12 |
| PLAU | PLTP | FABP7 | FABP3 | NEDD4 | ADIPOQ | VEGFA |
| ISG15 | LYZ | PLA2G2A | DMBT1 | IRF7 | CD14 | VCAM1 |
| NOS1 | SERPINA3 | CCL18 | CCR3 | CCL2 | CCR5 | CXCR4 |
| BLNK | FCGR2B | C3 | ACKR1 | GREM1 | IGF1 | IL17B |
| IL32 | IL33 | MDK | OGN | PDGFRL | SPP1 | C3AR1 |
| CRLF1 | CSF1R | CSF2RB | IL10RA | IL12RB2 | LGR5 | RXRG |
| ITGB2 | TYROBP | PTPRC | PRKCQ |  |  |  |

Table S2. The list of 67 immune-related DEGs.
